# Whole-Genome Sequence of the 1,4-Dioxane-Degrading Bacterium *Mycobacterium dioxanotrophicus* PH-06

**DOI:** 10.1128/genomeA.00625-17

**Published:** 2017-08-31

**Authors:** Ya He, Kangfei Wei, Kaiwei Si, Jacques Mathieu, Mengyan Li, Pedro J. J. Alvarez

**Affiliations:** a Department of Civil and Environmental Engineering, Rice University, Houston, Texas, USA; b International Academic Support and Delivery Unit, Beijing Genomic Institute, Shenzhen, Guangzhou, China; c Department of Chemistry and Environmental Science, New Jersey Institute of Technology, Newark, New Jersey, USA

## Abstract

We report here the complete genome sequence of *Mycobacterium dioxanotrophicus* PH-06, which is capable of using 1,4-dioxane as a sole source of carbon and energy. The reported sequence will enable the elucidation of this novel metabolic pathway and the development of molecular biomarkers to assess bioremediation potential at contaminated sites.

## GENOME ANNOUNCEMENT

*Mycobacterium dioxanotrophicus* PH-06 was isolated in South Korea from river sediment that had been contaminated with 1,4-dioxane (dioxane) for more than one decade (1). PH-06 utilizes dioxane, a groundwater contaminant of emerging concern, as its sole carbon and energy source. However, it does not harbor the well-studied monooxygenase gene cluster *thmADBC* that codes for the initiation of dioxane biodegradation in *Pseudonocardia dioxanivorans* CB1190 (2–4). Therefore, the genome sequence of PH-06 furthers our capability to discern novel dioxane biodegradation pathways and facilitates the development of biomarkers to assess dioxane bioremediation potential. Additionally, knowledge of the PH-06 genome broadens our understanding of the genus *Mycobacterium* and enables an assessment of PH-06 survival and performance in bioaugmentation applications.

PH-06 was grown in ammonium mineral salts medium (5) amended with 500 mg/L of dioxane and incubated at 30°C while shaking at 150 rpm. Cells were harvested during exponential growth, and genomic DNA was extracted using the UltraClean microbial DNA isolation kit (Mo Bio, Inc.). Whole-genome sequencing was conducted using both the PacBio RS II (Yale Center for Genome Analysis, http://ycga.yale.edu) and Illumina HiSeq 4000 (Beijing Genomic Institute [BGI], http://www.genomics.cn) platforms. The whole genome was assembled and annotated in collaboration with BGI as follows. First, prior to assembly, *k*-mer analysis was used to evaluate genome size, heterogeneity, and repeat information based on the data obtained by Illumina sequencing (6). Second, PacBio RS II reads were assembled using the RS_HGAP assembly of SMRT Analysis version 2.3.0 (https://github.com/PacificBiosciences/SMRT-Analysis) to obtain the main contig with a length close to the estimated genome size, and Illumina reads were used to correct and optimize the assembly results. Third, the contig’s bases were corrected with Quiver, Pilon, SOAPsnp, SOAPindel (http://soap.genomics.org.cn), and GATK (http://www.broadinstitute.org/gatk). Fourth, contig circle analysis was completed by verifying overlap regions. Fifth, Glimmer (7–9), TRF (10), RNAmmer version 1.2 (11), tRNAscan-SE version 1.3.1 (12), Infernal (13), Rfam (14), and BLAST were used to predict genes, repeat sequences, rRNAs, tRNAs, and noncoding RNAs (ncRNAs). Finally, predicted genes were analyzed against the GO (15), KEGG (16–19), COG (20), NR, Swiss-Prot (21), PHI (22), VFDB (23), ARDB (24), and CAZy (25) databases to annotate gene function and identify metabolic pathways, pathogenicity, and drug resistance.

The PH-06 genome consists of 4 contigs, including the chromosome (circular, 7.6 Mb), Plasmid_1 (circular, 156 kb), Plasmid_2 (circular, 153 kb), Plasmid_3 (linear, 106 kb), and Plasmid_4 (linear, 70 kb), and has an average G+C content of 66.46%. A total of 7,339 protein-encoding genes, 83 tRNAs, 9 rRNAs, and 4 ncRNAs are present. KEGG database analysis revealed genes encoding the complete citric acid and pentose phosphate pathways. Furthermore, 1,071 genes appear to be involved in the metabolism of xenobiotics. Pathogenicity analysis indicates that PH-06 harbors no known toxins or pathogenicity islands, suggesting it may be safe for bioaugmentation (22–24).

One gene cluster encoding putative propane monooxygenase is located on Plasmid_3. This gene cluster has high similarity to genes in (hydrocarbon-degrading) *Rhodococcus wratislaviensis* IFP2016 (89%) and *Mycobacterium chubuense* NBB4 (86%) (26, 27). Further studies are needed to determine the role of this gene cluster in dioxane biodegradation.

### Accession number(s).

The whole-genome sequence of *M. dioxanotrophicus* PH-06 has been deposited in GenBank under the accession numbers CP020809 to CP020813.
